# RETRACTION: The Efficacy of Low‐Frequency Ultrasound as an Added Treatment for Chronic Wounds: A Meta‐Analysis

**DOI:** 10.1111/iwj.70464

**Published:** 2025-04-02

## Abstract

**RETRACTION:** ChenH. , YuZ. , LiuN. , HuangJ. , LiangX. , LiangX. , M. LiangM., Li, and NiJ. , “The Efficacy of Low‐Frequency Ultrasound as an Added Treatment for Chronic Wounds: A Meta‐Analysis,” International Wound Journal 20, no. 2 (2023): 448–457, 10.1111/iwj.13893.35855676 PMC9885464

The above article, published online on 19 July 2022, in Wiley Online Library (http://onlinelibrary.wiley.com/), has been retracted by agreement between the journal Editor in Chief, Professor Keith Harding; and John Wiley & Sons Ltd. Following an investigation by the publisher, all parties have concluded that this article was accepted solely on the basis of a compromised peer review process. The editors have therefore decided to retract the article. The authors did not respond to our notice regarding the retraction.

**RETRACTION:** H. Chen , Z. Yu , N. Liu , J. Huang , X. Liang , X. Liang , M. Liang, M. Li, and J. Ni , “The Efficacy of Low‐Frequency Ultrasound as an Added Treatment for Chronic Wounds: A Meta‐Analysis,” International Wound Journal 20, no. 2 (2023): 448–457, 10.1111/iwj.13893.35855676 PMC9885464

The above article, published online on 19 July 2022, in Wiley Online Library (http://onlinelibrary.wiley.com/), has been retracted by agreement between the journal Editor in Chief, Professor Keith Harding; and John Wiley & Sons Ltd. Following an investigation by the publisher, all parties have concluded that this article was accepted solely on the basis of a compromised peer review process. The editors have therefore decided to retract the article. The authors did not respond to our notice regarding the retraction.

